# Isolation and Bioactivities of the Flavonoids Morin and Morin-3-*O*-β-D-glucopyranoside from *Acridocarpus orientalis*—A Wild Arabian Medicinal Plant

**DOI:** 10.3390/molecules191117763

**Published:** 2014-10-31

**Authors:** Javid Hussain, Liaqat Ali, Abdul Latif Khan, Najeeb Ur Rehman, Farah Jabeen, Jong-Sang Kim, Ahmed Al-Harrasi

**Affiliations:** 1 Department of Biological Sciences and Chemistry, College of Arts and Sciences, University of Nizwa, Birkat Al-Mouz, Nizwa 616, Oman; 2 UoN Chair of Oman’s Medicinal Plants and Marine Natural Products, University of Nizwa, Birkat Al-Mouz, Nizwa 616, Oman; E-Mails: malikhejric@gmail.com (L.A.); abdullatif@unizwa.edu.om (A.L.K.); najeeb@unizwa.edu.om (N.U.R.); fjabeen2009@yahoo.com (F.J.); 3 School of Food Science and Biotechnology, Kyungpook National University, Daegu 702-701, Korea; E-Mail: jongsangkim@gmail.com

**Keywords:** flavonoid, morin, *Acridocarpus orientalis*, Malpighiaceae, antioxidant, lipid peroxidation, cytotoxicity

## Abstract

*Acridocarpus orientalis* is an important medicinal plant for some of the locals of Arabian region. Very little is known about its phytochemical constituents. In the present study, we aimed to isolate bioactive chemicals from the crude methanolic extract of the aerial parts of *A. orientalis*. The extraction and isolation resulted in the purification of two flavonoids: morin (**1**) and morin-3-*O*-β-D-glucopyranoside (**2**). The structure elucidation was carried out by extensive analysis of spectroscopic data and comparison with the reported data for the known constituents. The pure isolates were subjected to various biological assays for their bioactivities. The compounds **1** and **2** were significantly active against the growth of various pathogenic fungi and phytotoxic against lettuce seed at higher concentrations. Furthermore, the free radical scavenging activities, anti-lipid peroxidation, and cytotoxic effects against HepG2, HT29, and HCT116 cancer cell lines were also assayed and the results are presented in this paper.

## 1. Introduction

Plants are the major source of active chemical constituents against diseases [1]. Most of the world’s population still relies on the folk medicines for the treatment of a large number of serious diseases. The biologically active principles of medicinal plants include flavonoids, phenolics, and polyphenols with promising anticancer and antioxidant activities [2,3]. *Acridocarpus orientalis* A. Juss. belongs to the family Malpighiaceae. This is a family of flowering plants found mainly in tropical, Mediterranean, and sandy regions of Asia, Africa and some other Gulf countries, including Oman [4,5]. Several *Acridocarpus* species are traditionally used as folk medicines all over the world. In addition to the medicinal advantages, some species have been reported to possess many ecological advantages as well. The plants of *A. socotranus* are also used in the traditional medicine system of Yemen as a cure for muscle pain and headaches [6,7]. *Acridocarpus chloropterus* leaves and stem are reported to have antiplasmodial, antileishmanial and antitrypanosomal activities and are found mainly in Tanzania [8]. *A. orientalis* has been reported mainly from the border areas of UAE and Oman, where it is used for the treatment of muscle pain, headaches, paralysis, tendon and joint pains as well as to treat the udder inflammation in cattle [6,7]. The use of *A. orientalis* against inflammatory diseases suggests further utility as a way to treat cancer [9], but *A. orientalis* has not been previously studied for its role against oxidative stress and cancerous cells.

Based on the above mentioned potential of the plant in the biological and pharmacological field, the present study was carried out in search for its bioactive chemical constituents. The methanolic extract and its various fractions were subjected to chromatographic separations that resulted in the isolation of two flavonoids for the first time from this plant. The structure elucidation and detailed biological evaluations of these compounds were then carried out and the results are presented in the present paper.

## 2. Results and Discussion

Compound **1** was purified from the ethyl acetate fraction in the form of a yellow colored amorphous powder. When the TLC plate was sprayed with ceric sulphate, it gave a yellow color, indicating the compound belongs to the flavone class. The presence of a flavonoid skeleton was also supported by UV maxima at 264 and 340 nm [10]. The IR absorptions were indicative of a hydroxyl functional group (3599 cm^−1^), aromatic functionalities (2922, 1591, and 1463 cm^−1^) and conjugated ketone (1665 cm^−1^) in the molecule. The EI-MS showed a molecular ion peak at *m*/*z* 302 [M^+^], along with the major fragments at *m*/*z* 153 and 149. These fragments appeared as a result of the *retro* Diels-Alder (RDA) fragmentation of the C ring [11].

**Figure 1:**
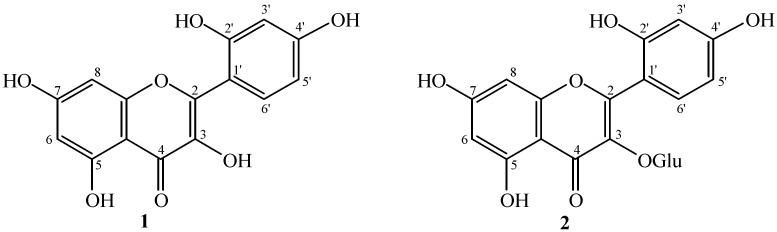
Structures of compound **1** and **2**.

The ^1^H-NMR spectrum of **1** exhibited signals at δ 6.19 and 6.36 (d, *J* = 2.0 Hz) which were indicative of the presence of *meta* coupled protons. These signals were thus assigned to H-6 and H-8, respectively. H-3', H-5', and H-6', protons of ring B appeared at δ 7.33 (1H d, *J* = 2.1 Hz), 6.90 (1H dd, *J* = 2.1, 8.1 Hz), and 7.30 (1H d, *J* = 8.1 Hz) respectively (Figure 1).

The ^13^C-NMR (BB and DEPT experiments) displayed ten quaternary and five methine carbons. The resonances at δ 99.8 and 94.7 were assigned to C-6 and C-8 respectively, while the ^13^C-NMR signals at δ 158.5, 163.3, and 163.2 were assigned to C-5, C-7, and C-9. These assignments indicated the ring A to be *meta* substituted with oxygenated groups [12]. The resonances at δ 149.8 and 159.3 were assigned to C-2' and C-4' positions respectively, while the remaining ring B carbons appeared at δ 116.9 (C-3'), 116.4 (C-5'), 122.9 (C-6'), and 122.8 (C-1'). The proposed structure was finally confirmed by the comparative analysis of the reported data for morin to that of compound **1** [13].

Compound **2** was isolated from the ethyl acetate fraction by repeated silica gel column chromatography. The structure was determined by the analysis of ^1^H and ^13^C-NMR data as well as by comparison with previously reported values [14,15]. MS showed a protonated molecular ion peak at *m/z* 465.2039 [M+1]^+^, suggesting the molecular formula C_21_H_20_O_12_. The fragment ion peak at *m/z* 303.3505 appeared due to loss of a deoxyhexose group. The IR spectrum showed absorptions for OH groups at 3450 cm^−1^. The ^1^H-NMR spectrum showed three aromatic proton signals at δ 7.83 (d, *J* = 2.4 Hz, H-3'), 6.87 (dd, *J* = 8.4, 2.4 Hz, H-5') and 6.86 (d, *J* = 8.4 Hz, H-6') in the form of an ABD spin-system suggesting a flavonol with a 2',4'- disubstituted B-ring. Ring A protons appeared as a pair of *meta* coupled proton signals at δ 6.19 (d, *J* = 2.4 Hz, H-6) and 6.39 (d, *J* = 2.4 Hz, H-8).

The ^13^C-NMR spectra supported this proposed structure and showed 21 signals including a carbonyl signal at δ 179.5 (C-4). It revealed chemical shifts at δ 135.7 (C-3), 163.0 (C-5), 166.2 (C-7), 145.8 (C-3'), 149.9 (C-4') for the oxygenated quaternary carbon centers. The signals at δ 105.3 (C-1''), 75.1 (C-2''), 77.2 (C-3''), 70.0 (C-4''), 73.1 (C-5''), and 61.9 (C-6'') were assigned to the glucose moiety in the molecule.

Thus, on the basis of the above discussions compound **2** was identified as morin-3-*O*-β-D-glucopyranoside. The identity of this compound was further substantiated by comparison of its spectral data with previously reported values [14,15].

### Anticancer, Allelopathic, Antifungal and Antioxidant Activities

**Figure 2:**
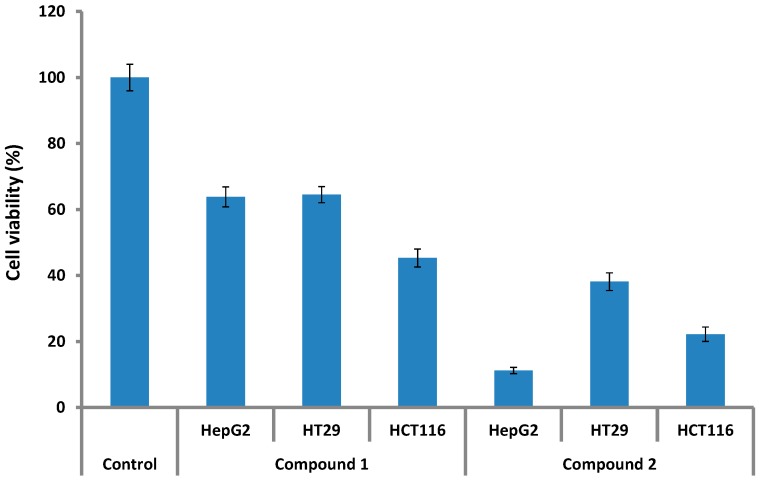
Anticancer cell activity of compounds **1** and **2**. The bars represent the mean values with standard error of three replicates.

Three cancer cell lines: colorectal adenocarcinoma (HT29), colorectal adenocarcinoma (HCT116); and human hepatoma derived cell line (HepG2) were used to analyze the cytotoxic potential of compounds **1** and **2**. Both the compounds were inactive against the HepG2, HCT116 and HT29 cancer cell lines at lower concentrations (0.1 to 50 ppm). However, when 100 ppm concentration of compound **1** was applied to the HepG2, HT29 and HCT116, the cancer cells cell viability was reduced appreciably to 63.8%, 64.5%, and 45.3%, respectively. Contrarily, compound **2** suppressed the cell viability of HepG2, HCT116 and HT29 cell lines to 11.20, 22.19 and 38.11% as compared to control (Figure 2).

**Figure 3:**
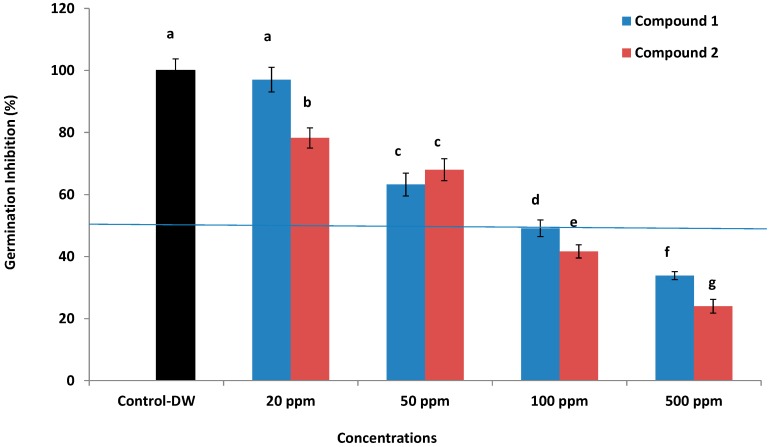
Allelopathic effects of compound **1** and **2** on the growth of lettuce seeds and pathogenic fungi; the bars represent the mean values with standard error of three replications. The different letter (a, b,...g) on each bar shows significantly (*p* < 0.05) different values as evaluated by DMRT analysis.

Flavonoids have been known to play an essential role in allelophathy as the plants release them through root exudation into the soil to safeguard it from pathogens and to act as food source for various soil microflora [16,17]. Both the compounds were also assessed for their allelopathic potential. Results showed that higher concentrations of 100 and 500 ppm significantly inhibit the growth and germination of lettuce seeds (Figure 3), whereby 100 and 500 ppm concentrations of compound **1** showed EC_50_ values of 49.21% and 33.82%, respectively, while in case of compound **2**, the same concentrations showed EC_50_ values of 38.97% and 24.87%, respectively. In the case of lower concentrations of 20 and 50 ppm neither compound showed an EC_50_ value, however, the effects of these concentrations were significantly inhibitive to lettuce growth as compared to control. Overall, the compound application showed inhibitive effects on seed germination and growth. This result is in conformity to the effects of other flavonoids like 7,8-benzoflavone [16]. Such an effect is reported for the first time for compounds **1** and **2**.

**Figure 4:**
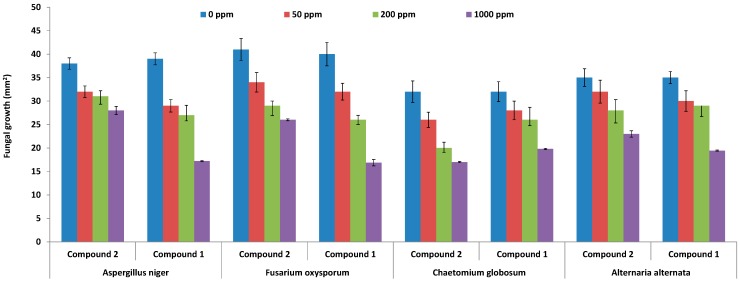
Antifungal activities of compound **1** and **2** against the growth pathogenic fungi. The bars represent the mean values with standard error of three replicates.

Compound **1** and **2** were also tested against known plant pathogens viz. *Aspergillus niger*, *Fusarium oxysporum*, *Chaetomium globosum*, and *Alternaria alternata*. The results showed that at 50 and 200 ppm concentrations of compound **1**, the fungi were actively growing, however, at 1000 ppm, compound **1** resulted in 17.21 ± 0.21, 16.87 ± 0.93, 19.437 ± 0.57 and 19.23 ± 0.74 mm^2^ of growth of *Aspergillus niger*, *Fusarium oxysporum*, *Chaetomium globosum*, and *Alternaria alternate*, respectively (Figure 4). Previously, Alam and Edziri *et al.* [18,19] have reported that various kinds of flavones can inhibit the growth of pathogenic fungi. In the present results, higher concentrations of compounds **1** and **2** reduced the growth of pathogenic fungi, suggesting the exudation via the root from JOA is allelopathic and antifungal, especially to *F. oxysporum*.

Both compounds were assessed for their potential antioxidant activities. The anti-lipid peroxidation results showed a very significant activity of compound **2** as compared with compound **1** (Table 1). Similarly, the potential of scavenging superoxide anion and DPPH radicals was significantly higher for compound **1** as compared to compound **2**. This was in conformity with the known standard ascorbic acid and quercetin. Flavonoids have been known to ameliorate oxidative stress, which develops inside cells during disease exposure [19,20]. Our results with compounds **1** and **2** also showed a strong potential in this regard, however, *in vivo* analysis would be needed to further elucidate the roles of these flavonoids.

**Table 1:** Antioxidant activities of isolated compounds.

Compounds	Anti-Lipid Peroxidation (%)	Superoxide Anion (%)	DPPH (%)
Control	92.34 ± 0.023a	64.19 ± 0.73a	98.88 ± 0.92a
Compound **1**	75.22 ± 0.023c	43.76 ± 0.0042b	97.96 ± 1.04a
Compound **2**	81.58 ± 0.12b	37.12 ± 0.18c	76.83 ± 0.78b

± shows the standard error of means of three replications. The analyses were performed with comparative standards. The different letter (a,b,c) in each column shows significant (*p* < 0.05) difference in the mean values with respect to control by DMRT analysis.

Previously some reports have suggested that morin or related derivatives/constituents showed a diverse range of biological functions. These compounds have been known to play essential roles in suppressing the growth of cancerous cells as shown by Tian *et al.* [21]. Similarly, Jin *et al.* [22], showed that morin suppress the growth of breast cancer cell lines by influencing the Akt pathway. Although, the role of morin (**1**) is somehow understood in HepG cells, the activity of morin-3-*O*-β-d-glucopyranoside (**2**) has not been previously known. The morin flavonoids also showed inhibitory effects towards seed germination of lettuce. In present study, our results showed that **1** and **2** also exhibited a similar pattern of growth diminishing effects. Previously, Munesada *et al.* [23] showed that flavonoid glycosides showed inhibitive effects for lettuce seed germination. Our results are in conformity with the results of Alam and Edziri *et al.* [18,19], however, compound **2** has not been known for its role against pathogenic fungi. In addition to that compounds **1** and **2** were assayed for their role in the enzyme inhibition of reverse transcriptase, protein-tyrosine kinase and xanthine oxidase, whilst it also showed anti-HIV, antiarteriosclerotic, and superoxide scavenging activities [22,23]. The present anti-oxidative stress results also showed that **1** and **2** are promising antioxidant agents. Natural products with such diverse biological and ecological roles suggest a wide range of applications for the benefit of humans.

## 3. Experimental Section

### 3.1. Plant Material

The plant, *Acridocarpus orientalis* A. Juss (Malpighiaceae), was collected from Al-Hamra, in the Ad-Dakhiliyah region of the Sultanate of Oman in March-April 2012, and has been identified by a plant taxonomist at the Department of Biological Sciences and Chemistry, University of Nizwa, Nizwa, Sultanate of Oman. The voucher specimen has been deposited in the herbarium of the department.

### 3.2. Extraction and Isolation

The air-dried ground plant material (*Acridocarpus orientalis*, 4.1 kg), was exhaustively extracted with 100% methanol (8 L) at room temperature. The extract was evaporated to yield a residue (600 g) that was partitioned in different solvents on the basis of increasing polarity to afford *n*-hexane (40.9 g), chloroform (84.8 g), ethyl acetate (30.6 g), and *n*-butanol (58.3 g) fractions. The ethyl acetate fraction (30 g) was subjected to column chromatography over a silica gel column (600 g, 70–230 mesh, Merck, Munich, Germany) using 10% ethyl acetate/*n*-hexane (2 × 500 mL) with a 5% gradient of increasing polarity up to 100% ethyl acetate, then by the gradient of methanol (1%, 2%, 5%, 10%, 20% and 40%), and finally washed with 100% methanol; twenty six fractions were thus collected. Two compounds were isolated using repeated column chromatography (flash silica gel, 230–400 mesh), and preparative TLC (silica gel 60 GF254), using ethyl acetate/*n*-hexane mixtures of various polarities. Fraction no. 5, obtained using 50% ethyl acetate/*n*-hexane was loaded on a silica gel column (flash silica 230–400 mesh) and eluted with gradients of ethyl acetate/*n*-hexane to purify compounds **1** [40.7 mg; methanol/chloroform (0.5:9.5)] eluted with 90% ethyl acetate/*n*-hexane and compound **2** [26 mg; methanol/ethyl acetate (1.5:8.5)] eluted with 4% methanol/ethyl acetate.

### 3.3. Anticancer Activities

Three cancer cell lines: colorectal adenocarcinoma (HT29), colorectal adenocarcinoma (HCT116); and human hepatoma derived cell line (HepG2) were used for testing the cytotoxicity of both compounds according to the method of Mosmann [24], and further modified by Kim *et al.* [25]. All cell lines were purchased from the ATCC (Manassas, VA, USA). Cell lines were cultured in Advanced DMEM supplemented with 10% inactivated NBCS and 5 mM L-glutamine, and grown at 37 °C in a humidified atmosphere of 5% CO_2_ in air. The results were generated from two independent experiments; each experiment was performed in triplicate by using a MTT [3-(4,5-dimethylthiazol-2-yl)-2,5-diphenyltetrazolium bromide] colorimetric assay.

### 3.4. Allelopathic and Antifungal Activities

The allelopathic potential of both compounds was studied by the method of Khan *et al.* [26]. Lettuce seeds (*Lectuca sativa*) were used to indicate the seed growth inhibition. EC_50_ values were also calculated which is the effective concentration of an extract/compound that induces 50% of inhibition of the tested organism [27]. Four different concentrations of 20, 50, 100, and 500 ppm of compound were prepared by dissolving it in 5% DMSO. A filter paper method was used. Fifteen lettuce seeds were placed on it and the dishes were sealed for incubation at 25 °C for 72 h. The experiment was repeated three times with three replicates.

Fungal pathogens viz. *Aspergillus niger*, *Fusarium oxysporum*, *Chaetomium globosum*, and *Alternaria alternate* were procured from the Leibniz-Institute DSMZ (Braunschweig, Germany). Antifungal activity was screened using a well-diffusion method [28]. Fungal growth was assessed against individual concentrations. Potato dextrose agar (PDA) plates were autoclaved at 121 °C for 15 min. Seven day-old fungal pads were grown on the PDA plates to serve as master plate and later as negative control. Two wells in each PDA plate were made by using a sterile weller. Three concentrations *i.e.*, 50, 200 and 500 ppm were prepared. The wells were loaded with different concentrations and fungal pads are transferred onto plates to assess the level of fungal growth inhibition. The plates were incubated for 5 days at 28 °C. Negative controls containing no sample were prepared too. Three replicates were maintained for each experiment, while the experiment was repeated twice.

### 3.5. Antioxidant Activities

Antioxidant activity of both the compounds was determined using the 1,1-diphenyl-2-picrylhydrazyl (DPPH) radical scavenging activity assay as reported by Gulati *et al.* [29]. To DPPH (50 μL of 0.1 mM; in methanol), 50 μL of compound (in a concentration range of 1.0 to 500 μg/mL) was mixed and kept in the dark at room temperature for 60 min. After incubation, the absorbance was recorded at 490 nm. The results were compared with the positive control (ascorbic acid). The antioxidant activity was expressed as percentage (%) inhibition = (Ac − As/Ac) × 100; where A_C_ is absorbance of control and A_S_ is the absorbance of sample. The potential of compounds to inhibit the extent of lipid peroxidation was assessed through a modified thiobarbituric acid reactive substances (TBARS) method [30]. This was based on the peroxidation of a liposome (phosphatidyl-choline 50 mg/mL) induced by iron chloride (200 µL, 1 mM) containing potassium chloride (300 mM) in the presence of both compounds (50 µL). Peroxidation was initiated by ascorbate (125 µL with 0.16 mM) and the reaction mixture was incubated for 30 min at 37 °C. A mixture of trichloroacetic acid (0.75 mL with 1.5:1 (v:v)) and TBA (0.38%) was added to the reaction mixture. It was kept in boiling water for 30 min until a pink color appeared. The production of TBARS, mainly malonaldehyde, as a secondary product of peroxidation, was measured at 535 nm. A control without compound was used as blank. Inhibition was calculated using the expression (IP%): (1 − At/Ao) × 100; where At and Ao are compound and control absorbance after incubation for 30 min. The experiment was repeated three times. Superoxide radical scavenging activity was measured by reducing nitroblue tetrazolium (NBT) as reported by Hazra *et al.* [31]. The non-enzymatic phenazine methosulfate-nicotinamide adenine dinucleotide (PMS/NADH) system generates superoxide radicals, which reduce NBT to a purple formazan. The 1 mL reaction mixture contained phosphate buffer (20 mM, pH 7.4), NADH (73 μM), NBT (50 μM), PMS (15 μM) and compounds (20 μg/mL). After incubation for 5 min at room temperature, the absorbance at 562 nm was measured against blank to determine the quantity of formazan generated. All tests were performed three times. Quercetin was used as positive control.

## 4. Conclusions

Local communities of the Arabian region have used *Acridocarpus orientalis* for medicinal purposes. This suggests the presence of bioactive chemical constituents. We isolated and identified two flavonoids: morin (**1**) and morin-3-*O*-β-D-glucopyranoside (**2**) from the methanolic extract. The compounds showed antifungal, phytotoxic, anticancer and anti-lipid peroxidation properties.
